# Dietary management for pyridoxine‐dependent epilepsy due to α‐aminoadipic semialdehyde dehydrogenase deficiency, a follow‐on from the international consortium guidelines

**DOI:** 10.1002/jmd2.12418

**Published:** 2024-04-03

**Authors:** Marjorie Dixon, Chloe Millington, Laurie Bernstein, Curtis R. Coughlin, Morgan Drumm, Sommer Gaughan, Clara D. M. van Karnebeek, Annemiek M. J. van Wegberg

**Affiliations:** ^1^ Dietetics Department Great Ormond Street Hospital for Children NHS Foundation Trust London UK; ^2^ Section of Clinical Genetics and Metabolism, Department of Pediatrics University of Colorado Anschutz Medical Campus Aurora Colorado USA; ^3^ Emma Center for Personalized Medicine, Departments of Pediatrics and Human Genetics, Amsterdam Gastroenterology Endocrinology and Metabolism Amsterdam Univeristy Medical Center Amsterdam The Netherlands; ^4^ Department of Gastroenterology and Hepatology‐Dietetics Radboud University Medical Center Nijmegen The Netherlands

**Keywords:** diet, lysine, management, PDE‐ALDH7A1, pyridoxine‐dependent epilepsy

## Abstract

Pyridoxine‐dependent epilepsy (PDE‐ALDH7A1) is a neurometabolic disorder in the lysine metabolism pathway. In 2014 and 2021, the International PDE consortium published consensus guidelines about diagnosis and management. In this follow‐on, a literature review was performed and nutrition management was evaluated through an international dietary questionnaire with 40 respondents. This manuscript discusses consensus dietary statements and the practical provision of lysine reduction therapies. Results from the questionnaire, statements from the PDE consensus guidelines, new data from the literature, as well as clinical practice experience of the metabolic dietitian group form the basis of these updated practical diet recommendations. These dietary management recommendations can support dietitians, nutritionists, and physicians in initiation and monitoring of lysine reduction therapies for PDE‐ALDH7A1 patients and families.

**Synopsis**

These dietary management recommendations provide guidance with practical examples on initiation, on‐going management and monitoring of lysine reduction therapies for different age groups of children.

## 1 INTRODUCTION

**FIGURE 1:**
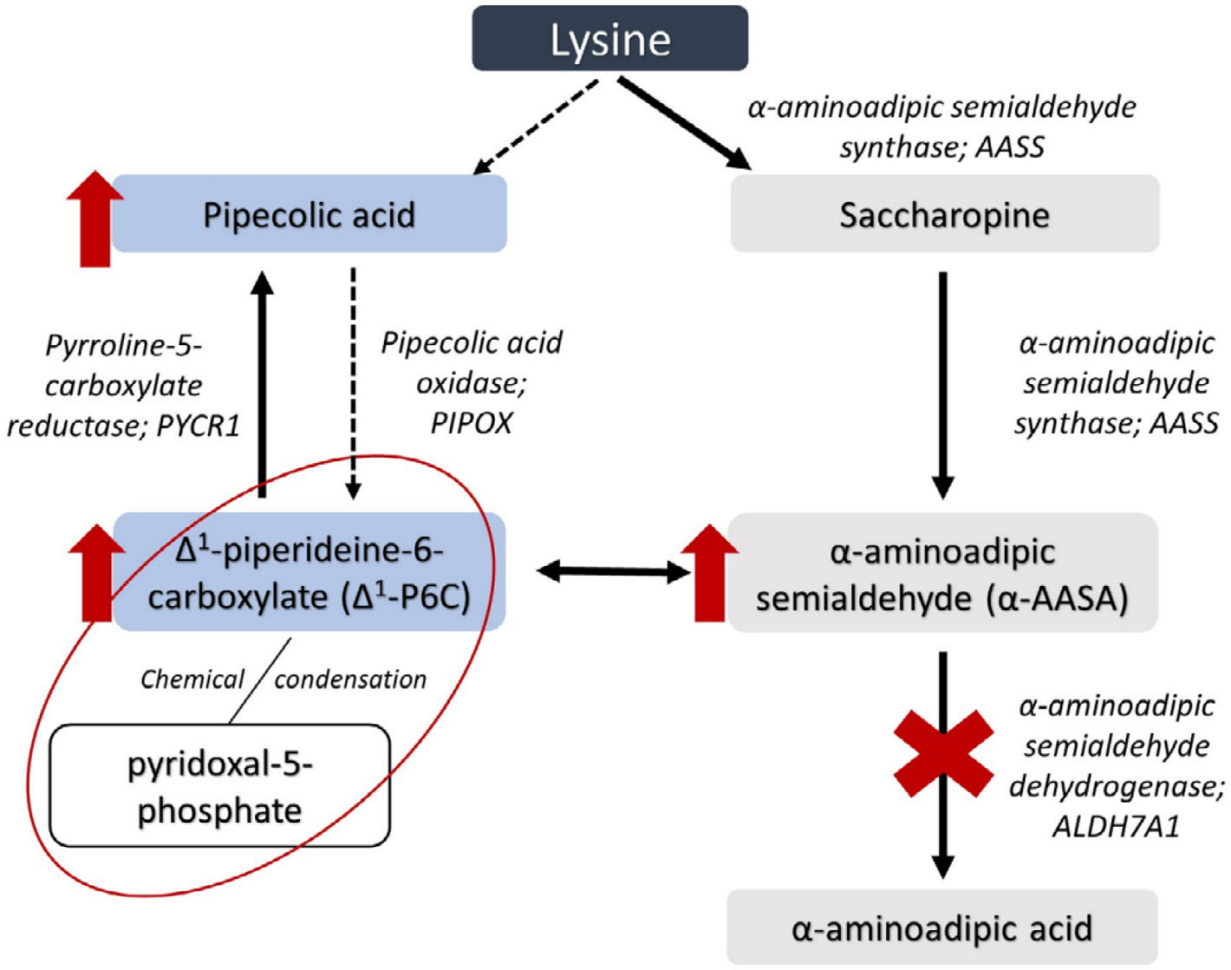
Lysine metabolism and pyridoxine‐dependent epilepsy.^4^ Pyridoxine‐dependent epilepsy is caused by the deficiency of α‐aminoadipic semialdehyde (α‐ASSA) dehydrogenase, which results in the accumulation of multiple metabolites including α‐AASA, Δ^1^‐piperideine‐6‐carboxylate (Δ^1^‐P6C) and pipecolic acid.

Pyridoxine‐dependent epilepsy (PDE‐ALDH7A1; OMIM 266100) is a neurometabolic disorder in the lysine metabolism pathway with an incidence of 1:65 000 to 250 000 live births.^1^, ^2^ Bi‐allelic pathogenic variants in *ALDH7A1* (also referred to as antiquitin) cause a deficiency of α‐aminoadipic semialdehyde (α‐AASA) dehydrogenase,^3^ Figure 1. This enzymatic defect results in the accumulation of several metabolites: α‐AASA,^5^ Δ^1^‐piperideine‐6‐carboxylate (Δ^1^‐P6C),^6^ pipecolic acid^7^ and the recently discovered biomarkers 2S,6R‐oxopropylpiperidine‐2‐carboxylic acid^8^ and 6‐oxo‐pipecolate.^9^ Lysine does not accumulate.

Secondary increased levels of Δ^1^‐P6C deactivate pyridoxal‐5‐phosphate (PLP), the active form of pyridoxine, which is a cofactor for over 160 enzymic activities.^10^ Depletion of PLP causes an epileptic encephalopathy,^11^ which can be fatal.^12^, ^13^, ^14^

PDE patients classically present neonatally with epilepsy refractory to conventional anti‐epileptic medications but are responsive to pyridoxine supplementation. Some may also present after infancy or even during adolescence.^15^, ^16^, ^17^, ^18^ Currently, PDE is not included in newborn screening programmes although it is being considered.^19^

Treatment with pyridoxine overcomes the depletion of PLP, as seizures are well controlled in roughly 90% of patients.^15^ However, despite both early treatment initiation and seizure control, 75% of PDE patients show global developmental delay and/or intellectual disability.^15^, ^20^ This is probably related to one or multiple of the accumulated biomarkers, but it is not yet fully understood. In an effort to improve outcome, two newer therapies have been introduced in addition to pyridoxine to limit production of accumulated biomarkers. A lysine‐restricted diet (LRD) is implemented to reduce the accumulation of lysine‐derived substrates.^21^ And L‐Arginine supplementation because it competes with lysine transport at the gut, at the mitochondrial membranes (ornithine carriers ORNT1 and ORNT2) and the blood brain barrier (cationic amino acid transporter 1).^22^ These additional therapies are termed double or triple therapy and most recently lysine reduction therapy (LRT).

Although the evidence was limited, the first recommendations for a LRD were published by the International PDE consortium (www.pdeonline.org) in 2014 to aim for a standardised approach.^23^ In addition, the international PDE registry was established to understand the effect of current therapies and better unravel PDE disease mechanisms. The registry now includes over 180 patients (www.pdeonline.org). The PDE consortium consensus guidelines for the diagnosis and treatment of PDE‐ALDH7A1 patients published in 2021^4^ updated the Stockler et al.^24^ diagnosis and standard treatment and van Karnebeek et al.^23^ LRD recommendations. The PDE consortium guideline 2021 includes 20 statements related to the general principles of treatment, treatment monitoring and emergency treatment. It does not discuss the practical application of the LRD as described by van Karnebeek et al. The statements were classified according to the GRADE quality of evidence; high (level A), moderate (level B), low (level C) or very low (level D).

The current study was initiated in 2021 to address the knowledge gaps in dietary management. This manuscript aims to review the literature and update information on LRD regarding the new 2021 statements and the 2014 recommendations. It also provides practical examples on provision of LRD. Finally, we provide novel 2023 international dietary management recommendations for PDE‐ALDH7A1.

## 2 METHODS

### 2.1 Dietary management recommendations procedure

These dietary management recommendations were developed by an international group of metabolic dietitians (M.D., C.M., L.B, S.G. and A.M.J.v.W.) in conjunction with the international PDE consortium (C.v.K. and C.R.C.). A questionnaire was developed to understand more about individual practice in the dietary prescription for children and adults with PDE. Results from the questionnaire, statements from the PDE consensus guideline 2021,^4^ recommendations from van Karnebeek et al.,^23^ new literature review and summary plus clinical practice experience of the metabolic dietitian authors form the basis of these updated practical diet recommendations. Full agreement was reached among the authors for all dietary recommendations.

### 2.2 Questionnaire development

A web‐based questionnaire was developed using the online software SurveyMonkey© (www.surveymonkey.com). The questionnaire consisted of closed and open‐ended questions. Using adaptive questioning, participants answered 4–42 questions. For several questions, multiple answer options could be given. The questions with multiple answers options are marked with an asterisk (*) in the result section.

Before distribution the content and technical functionality were reviewed by independent colleagues. Informed consent was not required, as no identifiable patient data were requested.

Genetic Metabolic Dietitian International (GMDI) Metab Listserve, Society for the Study of Inborn Errors of Metabolism Dietitians Group (SSIEM‐DG) and British Inherited Metabolic Disease Dietitians Group (BIMDG) were contacted in January 2020 to invite those managing PDE patients with LRD to sign up to receive the questionnaire. People were also requested to circulate among their country's national e‐mail lists (snow‐ball sampling). To avoid duplicate entries, it was requested that only one team member per centre sign up.

Data were collected from 6 April to 17 May, 2021. IP addresses, general information and the first set of questions were checked for double entries from the same individual. Data were analysed in SPSS version 25. Only descriptive data are presented.

## 3 LITERATURE OVERVIEW OF TREATMENT OUTCOME OF LYSINE REDUCTION THERAPIES

To date, 43 patients treated with both pyridoxine and LRT have been described in 17 case reports or case series. These manuscripts use several outcome measures, which limits the utility of a meta‐analysis. In general, patients treated with LRT are reported to have improvements in seizure frequency, behaviour, psychomotor development, speech and biomarkers associated with PDE‐ALDH7A1 (Table 1).^14^, ^21^, ^25^, ^26^, ^27^, ^28^, ^29^, ^30^, ^31^, ^32^, ^33^, ^34^, ^35^, ^36^, ^37^, ^38^, ^39^, ^40^, ^41^

**TABLE 1:** Patients treated with lysine reduction therapies.

Publication(s)	No. of subjects	Age (years)	Treatment	Outcomes reported
Case report or case series				
van Karnebeek et al.^21^	7^a^	0.4–12.3	B6 + LRD	Biomarker,^e^ development, sz control
Mercimek‐Mahmutoglu et al.^25^	1	12	B6 + arginine	Biomarker, development
Mercimek‐Mahmutoglu et al.^26^; Mahajnah et al.^27^	1^b^	3.6	B6 + LRD	Biomarker,^e^ development
Coughlin et al.^28^	6^a^	2–8.5	B6 + arginine + LRD	Biomarker,^e^ development, seizure (sz) control
Yuzyuk et al.^29^	2^b^	2.6–3.6	B6 + arginine + LRD	Biomarker,^e^ clinical
Al Teneiji et al.^30^	5	0.6–17	B6 + arginine	Clinical,^e^ biomarkers
	4^c^	1–10	B6 + arginine + LRD	Clinical,^e^ biomarkers
Coci et al.^31^	1	1.25	B6 + arginine	Brain imaging,^e^ clinical
de Rooy et al.^32^	1^d^	4	B6 + LRD	Development^e^
Navarro‐Abia et al, 2018^33^	1	4	B6 + arginine + LRD	Brain imaging,^e^ clinical
Toldo et al.^14^	1	1.5	B6 + arginine + LRD	Brain imaging,^e^ clinical
Kava et al.^34^	1	4	B6 + LRD	Clinical, sz control
Minet et al.^35^	1	3	B6 + arginine + LRD	Clinical,^e^ sz control, biochemical
Strijker et al.^36^	7^d^	1–14	B6 + arginine + LRD	Development,^e^ clinical
	5	6–13	B6 + arginine	
Tseng et al.^37^	1	28	B6 + arginine	Development^e^
Tseng et al.^38^	3^b^	2.9–9	B6 + arginine + LRD	Development^e^
Kim et al.^39^	1	0.03	B6 + arginine + LRD	Biomarkers^e^
Cohort studies comparing timing of treatment and treatment modalities (B_6_ monotherapy compared to B_6_ + LRT)				
Tseng et al.^40^	20	3–38.7	B6 monotherapy	Clinical,^e^ development
	17	6–22.1	B6 + arginine (*n* = 2); B6 + LRD (*n* = 2); B6 + arginine + LRD (*n* = 13)	
Coughlin et al.^41^	45	0.3–41.0	B6 monotherapy	Development,^e^ clinical
	55	0.3–27	B6 + LRT	

Abbreviations: LRD, lysine‐restricted diet; LRT, lysine reduction therapies; No., number; sz, seizure.

^a^ Three subjects were reported after treatment with B_6_ + lysine‐restricted diet and again after adding arginine supplementation.

^b^ One subject was reported in both publications.

^c^ One subject was first reported after treatment with B_6_ + lysine‐restricted diet and again after adding arginine supplementation.

^d^ One subject was first reported after treatment with B_6_ + lysine‐restricted diet and again after adding arginine supplementation.

^e^ Primary outcome.

Two recent studies from the PDE registry data were published. Coughlin et al. reported the effect of LRT to the standardised developmental testing scores of 60 patients. In total 112 developmental testing score results were evaluated, 50 (44.6%) when patients were treated with pyridoxine monotherapy and 62 (55.4%) with any additional form of LRT. The median age of LRT initiation was 4.9 years of life (interquartile range [IQR] 0.87–10.2 years). After correcting for various factors they found a moderate increase of 6.9 (95% confidence interval [CI]: −2.7 to 16.5) points on developmental testing. In a second analysis, only patients on treatment before the age of 6 months (*n* = 8) were included. Analysis showed a significant increase of 21.9 (95% CI: 1.7–42.0) points on developmental testing suggesting treatment initiation at a young age is beneficial.^41^ Tseng et al. compared the neurodevelopmental outcomes of siblings with a difference in age at treatment initiation. They compared siblings within nine families with pyridoxine monotherapy and siblings within another nine families on pyridoxine combined with any form of LRT.^40^ Between siblings on pyridoxine monotherapy, no differences were apparent. For families on LRT, most early treated siblings performed better than their older siblings in overall neurodevelopment, cognition, fine motor and behaviour/psychiatry, confirming the findings of Coughlin et al.

## 4 QUESTIONNAIRE RESPONDENTS AND THEIR PATIENTS

### 4.1 Questionnaire results: respondents characteristics

A total of 45 respondents completed the SurveyMonkey© questionnaire, 40 were analysed. There was one duplicate entry, three only answered the general information and/or patient characteristics and one did not treat any PDE patients.

Thirty‐seven respondents (93%) are dietitians/nutritionists and three neurologist/neurology consultants (8%). The respondents originated from 13 countries located in 4 continents (Asia *n* = 2; Europe *n* = 15; North America *n* = 20; Australia/Oceania *n* = 3).

### 4.2 Questionnaire results: patient characteristics and treatment

Respondents managed 146 children and 9 adults with PDE: most were managed in Europe (*n* = 77; 50%) and North America (*n* = 58; 37%) (Table S1). Each centre managed between 1 and 22 PDE patients, with six treating eight patients or more.

Patients were treated with triple therapy of pyridoxine, LRD and arginine (*n* = 72; 46%), double therapy of either pyridoxine and LRD (*n* = 33; 21%) or pyridoxine and arginine (*n* = 21; 14%), monotherapy of pyridoxine (*n* = 27; 17%) or LRD (*n* = 2; 1%). A lysine‐free protein substitute was prescribed to 81% (*n* = 87/107) of patients on a LRD.

## 5 CONSENSUS RECOMMENDATIONS

*The PDE consortium consensus guidelines 2021 state* ^4^:

- LRT have been associated with improved long‐term neurologic outcomes. Therapies include pyridoxine supplementation in combination with a LRD or arginine supplementation and a combination of all three (statement 10, level of evidence: C).
- All newborns and infants with PDE should be treated with LRT (statement 12, level of evidence: D).
- All children and adolescents and adults should be offered treatment with LRT. When there is cognitive delay, behavioural difficulties or poor seizure control, they should be treated with LRT (statement 20 and 21, level of evidence: D).

### 5.1 Principles of lysine reduction therapies

#### 5.1.1 Lysine restriction/natural protein restriction and diet calculation

*Questionnaire results*: to devise the LRD 79% (*n* = 30/38) used the PDE consortium recommendations 2014,^23^ 24% (*n* = 9/38) GA1 guideline 2017,^42^ 5% (*n* = 2/38) Food and Agriculture Organization (FAO)/World Health Organization (WHO)/United Nations University (UNU) 2007,^43^ 3% (*n* = 1/38) local/country recommendations and 11% (*n* = 4/38) none of these (Table S5—Q15*).

Diet prescription for patients was calculated using different methods. Half the respondents (*n* = 19/38) used a combination of lysine and protein, 37% (*n* = 14/38) protein only, 3% (*n* = 1/38) lysine only and 11% (*n* = 4/38) used another method (Table S6—Q16).

*Discussion and clinical practical experience*: lysine is an essential amino acid and is proteogenic and has a role in production of carnitine and calcium uptake, therefore lysine requirements should be met. The FAO/WHO/UNU 2007 provides lysine requirements for infants, children and adolescents.^43^ The PDE consortium guidelines 2014 provide age‐dependent lysine‐restriction ranges (mg/kg bodyweight), (Table 2). van Karnebeek et al., use the terminology, lysine‐restriction (mg/kg/bodyweight) rather than requirements.^23^ These lysine‐restrictions were based on the GA1 guidelines 2011,^45^ Yannicelli^46^ plus the FAO/WHO/UNU 1985 guidelines^47^ and then adapted for PDE based on experience of the consortium. The GA1 guidelines for lysine are based on earlier clinical experience by Muller et al.^48^ and the protein requirements by Dewey et al.^49^ The latter was used to update the FAO/WHO/UNU 1985 amino acid requirements,^47^ the subsequent 2007 publication provided lysine requirements for more ages.^43^ There are some differences between the PDE consortium guidelines 2014,^23^ the FAO/WHO/UNU 2007^43^ and revised GA1 guidelines 2023^44^ lysine requirements, which need to be considered (Table 2). The PDE consortium provides lysine‐restrictions for different age groups as a range, whereas the FAO/WHO/UNU 2007 and GA1 guidelines (for infants) provide mostly as a single figure.

**TABLE 2:** Recommended lysine‐restriction ranges, lysine requirements, protein equivalent intakes and safe level of protein intake in infants, children and adolescents.

Age	PDE Lysine‐restriction mg/kg/day^23^	FAO/WHO/UNU lysine requirements mg/kg/day^43^	GA1 lysine from natural protein mg/kg/day^44^	GA1 protein equivalent g/kg/day from lysine‐free protein substitute^44^	FAO/WHO/UNU Safe level of protein intake g/kg/day^43^
1 month	70–100	119^a^	100	1.3–0.8	1.77
2 months	70–100	87^a^	100	1.3–0.8	1.5
3 months	70–100	75^a^	100	1.3–0.8	1.36
4 months	70–100	68^a^	100	1.3–0.8	1.24
<0.5 years	70–100	65 (5 months)^a^	100	1.3–0.8	
0.5 to <1.0 years	55–70	64 (6 months)	90 (7–12 months)	1.0–0.8 (7–12 months)	1.14 at 0.5 years 1.31 weaned infant
1 to <4 years	50–80	45 (1–2 years)	80–60 (1–3 years)	0.8	1 years—1.14 1.5 years—1.03 2 years—0.97 3 years—0.9
4 to <7 years	40–70	35 (3–10 years)	60–50 (4–6 years)	0.8 (4–6 years)	4 years—0.86 5 years—0.85 6 years—0.89
7 to <11 years	35–65	35 (3–10 years)			7 years—0.91 8 years—0.92 9 years—0.92 10 years—0.91
Female					
11 to <15 years	35–40	35 (11–14 years)			11 years—0.9 12 years—0.89 13 years—0.89 14 years—0.87
15 to <19 years	33–40	33			15 years—0.85 16 years—0.84 17 years—0.83 18 years—0.82
>19 years	30–40	30			
Male					
11 to <15 years	35–40	35 (11–14 years)			11 years—0.91 12 years—0.90 13 years—0.90 14 years—0.89
15 to <19 years	33–45	33 (15–18 years)			15 years—0.88 16 years—0.87 17 years—0.86 18 years—0.85
>19 years	30–40	30 (>18 years)			

Abbreviation: PDE, pyridoxine‐dependent epilepsy.

^a^ Lysine intake of exclusively breast fed infant to be used as the best estimate of requirements for this age group. It should be recognised this may be more generous compared to actual demands. From 6 months, the factorial method is used, FAO/WHO/UNU 2007.

The PDE consortium guidelines 2014 age‐dependent lysine‐restriction ranges (Table 2) are recommended to guide lysine intake.^23^ Over restriction of lysine intake has the potential risk of malnutrition.^4^ Dietetic experience from the group considers the lower end of the lysine‐restriction ranges to be too low for some infants and young children as evidenced by low plasma lysine levels and advise providing the upper end of the age‐dependent ranges (Table 2). Experience of diet in older children with PDE is limited, the mid to upper end of the lysine‐restriction ranges may also be necessary to achieve optimal growth particularly during puberty and particularly if no protein substitute is given. Close monitoring of diet through regular dietary assessment, growth and quantitative plasma amino acids is advised and detailed in Section 5.4.1.

Questionnaire respondents rarely calculate the diet based on lysine alone and most use protein and lysine or protein. Many countries' food databases lack precise or recent analysis of the lysine content of food making it very difficult to accurately prescribe a LRD. The amount of lysine per gram of protein varies widely, from 30 to 90 mg per gram of protein (Table 3). The lysine in meat, fish and dairy foods is two‐ to three‐fold higher than cereals, rice, fruit and vegetables and the portion size per 1 g of protein is much smaller. Therefore, a diet based on the lysine content of foods is more accurate than using protein values. If lysine food composition data are not available, the lysine intake from different food types can be estimated using Table 3. Practical details are provided in Section 5.3.1. The total daily natural protein intake will vary depending on the food sources to provide lysine.

**TABLE 3:** Lysine content of selected high and low biological protein foods.

High biological value protein foods (HBV)	Lysine mg per 1 g protein	Weight of food (g) to provide 1 g of protein^a^
Fish	90	4–6
Meat	70–90	3–5
Cheese	70–80	4–5
Legumes	70	11–20
Milk	70	30
Yoghurt	70	14–25
Soya beans	60	7
Eggs	60	8
Cashew nuts	51	6
Nuts	33	4–12

Low biological value protein foods (LBV)	Lysine mg/g protein	Weight of food (g) to provide 1 g of protein
Cooked potato	60	53
Cooked vegetables	50	30–40
Cereals/baked goods with egg or milk	50	14–18
Rice/barley/oats	40	9–36
Bread without egg/milk	30	9–11
Cooked pasta without egg/milk	30	20–30

*Note*: Adapted from: Kölker et al.^50^ and Finglas et al.^51^ and the MetabolicPro analysis programme (www.metabolicpro.org).

^a^ This provides a guide to the typical weight of food to provide 1 g of protein. It is advised to use 1 g protein exchange lists used in your own centre.

*Recommendation*: for accuracy, it is recommended to count or estimate lysine content of food to ensure an adequate intake and reduce daily variability.

#### 5.1.2 Total protein requirements and provision of lysine‐free protein substitute

*The PDE consortium consensus guidelines 2021 state* ^4^:

- In newborns and infants, a LRD should include a lysine‐free protein substitute to maintain adequate total protein and micronutrient intake and a low‐normal plasma lysine level (statement 13, level of evidence: D).
- In children and adolescents, a LRD may include a lysine‐free protein substitute. If the lysine‐free protein substitute is not well tolerated lysine reduction may be achieved by reducing total natural protein to the low end of age‐appropriate needs (statement 17, level of evidence: D).

*Questionnaire results*: to devise the diet plan for total protein 71% (*n* = 27/38) used PDE consortium guidelines 2014,^23^ 37% (*n* = 14/38) GA1 guidelines,^42^ 11% (*n* = 4/38) local/country recommendations and 16% (*n* = 6/38) none of these (Table S5—Q15*).

Most respondents (*n* = 32/38; 84%) prescribe a lysine‐free protein substitute and 11% (*n* = 4/38) only in some patients (Table S8—Q18).

*Discussion and clinical practice experience*: the PDE consortium 2014 provides age‐dependent recommendation ranges for total protein (natural protein plus lysine‐free protein substitute) as g/kg/day. These figures were based on 130%–135% of the patient's age‐appropriate dietary reference intake (DRI).^52^ The rationale for these recommendations being greater than the DRI is the rapid digestion and absorption of free amino acids provided in the lysine‐free amino acid supplement and subsequent decreased nitrogen retention.^53^, ^54^, ^55^ However, the total protein figures recommended by van Karnebeek in Table 1, appear to be DRI for natural protein *plus* 130%–135% DRI from the lysine‐free amino acid‐based formula.^23^ As such, the upper end of these age‐dependent total protein ranges seems relatively high in comparison to recommendations for other inherited metabolic disorders on similar diets such as the phenylalanine‐restricted diet for phenylketonuria^56^, ^57^ or the LRD for GA1^50^ particularly for under 1 year of age.

In comparison, the recent revisions of the GA1 guidelines,^42^, ^44^ do not give total protein recommendations as the authors recognise this will vary depending on the source of natural protein foods to provide the lysine requirement. A diet which provides the lysine requirement mainly from low biological value (LBV) protein foods will be much higher in natural protein compared to a combination of high biological value (HBV) and LBV protein foods. Instead, Boy et al. make age‐dependent recommendation ranges for protein equivalent g/kg/day from protein substitute (until 6 years of age) (Table 2) to ensure adequate supply of essential amino acids and vitamins, minerals and trace elements. These figures are comparable with the safe level of protein intake for age.^43^ A similar approach seems appropriate to adopt for provision of protein equivalent g/kg/day in PDE to ensure a safety net when natural protein intake is variable and as a source of vitamins, minerals and arginine. It would appear prudent to prescribe a protein substitute at least for infants and young children who are growing rapidly as lysine requirements alone may not provide adequate protein. Although it is currently unclear what the optimal total protein intake should be, there is general agreement that this should provide no less than the safe levels of protein intake for age.^43^

*Recommendation*: infants and young children on LRD should be prescribed a protein‐substitute. Total protein intake should at least provide the safe level of protein intake for age,^43^ Table 2.

For older children and adolescents, the PDE consortium 2021 advises the diet can be achieved without a lysine‐free protein substitute (if not well tolerated) by reducing total natural protein to the low end of age‐appropriate needs.^4^ Interestingly, this is described as a protein restriction rather than lysine. If a protein substitute is not given, we recommend the lysine requirements (Table 2) provide at least the safe level of protein intake for age.^43^ This is met if the lysine intake is provided at the mid to upper end of the PDE consortium lysine‐restrictions for age and from a combination of 70% LBV and 30% HBV protein foods (Supplement S2). A micronutrient supplement is essential as intakes of some are likely to be low such as vitamin B12, iron and calcium.

For some older children continued use or introduction of a protein substitute may be warranted to ensure on‐going nutritional adequacy of the LRD for growth and development. No specific age‐related recommendations are provided as this needs to be individualised to the child's needs considering the natural protein intake from lysine sources.

Important clinical outcome measures to assess for nutritional adequacy are growth, development, plasma amino acid profiles and general health.

*Recommendation*: older children and adolescents on LRD with no protein substitute should have at least the safe level of protein intake for age,^43^ Table 2 and a micronutrient supplement.

#### 5.1.3 Practical aspects of lysine‐free protein substitute

*Questionnaire results*: a lysine‐free, tryptophan low protein substitutes specific for glutaric aciduria type 1 (GA1) is mostly prescribed (*n* = 35/36; 97%) (Table S9—Q19*). The amount of lysine‐free protein substitute given was based on either the PDE consortium guidelines 2014^23^ (*n* = 27/38; 71%), GA1 guidelines 2017^42^ (*n* = 14/38; 37%), local/country recommendations (*n* = 4/38; 11%) or none of these (*n* = 6/38; 16%) (Table S5—Q15*). The frequency of giving protein substitute was: four times per day (*n* = 4/38; 12%), three times a day (*n* = 21/38; 55%), twice a day (*n* = 11/38; 29%) or once per day (*n* = 2/38; 6%) (Table S12—Q22*).

*Discussion and clinical practice experience*: few PDE‐specific protein substitutes exist so GA1 protein substitutes which are lysine and tryptophan free are commonly used. Tryptophan restriction is not required in PDE, therefore the diet and lysine‐free protein substitute need to provide an adequate intake. Tryptophan is discussed in Section 5.3.6.

The PDE consortium does not give advice about the frequency of administration of the lysine‐free protein substitute. Standard practice and rationale used for amino acid disorders on similar diets can be adopted as described by MacDonald et al.^58^ To enhance utilisation, the protein substitute should ideally be given two to three times a day with main meals. The exact frequency and timing will also depend on the total amount prescribed, practical feasibility and be individualised to the child.

*Recommendation*: adequate tryptophan needs to be provided from protein substitute and or diet. Protein substitutes should be administered two to three times/day with natural protein foods.

#### 5.1.4 Energy requirements

*Questionnaire results*: to plan daily energy intake 55% (*n* = 21/38) used the PDE consortium recommendations 2014,^23^ 6% (*n* = 10/38) GA1 recommendations 2017,^42^ 26% (*n* = 10/38) local/country recommendations, 29% (*n* = 11/38) FAO/WHO/UNU recommendations and 8% (*n* = 3/38) none of these (Table S5—Q15*).

*Discussion and clinical practice experience*: the PDE consortium 2014 suggested age‐dependent energy intakes as kcal/kg/day,^23^ only 55% used these. This is not unexpected as these are set much higher than other population recommendations such as FAO/WHO/UNU.^59^ In clinical experience, energy requirements in PDE appear to be the same as the general population.

*Recommendation*: international or national energy requirements for the general population are recommended.

#### 5.1.5 Micronutrient requirements

*Questionnaire results*: this topic was not included in the questionnaire.

*Discussion and clinical practice experience*: requirements for micronutrients, except pyridoxine, are considered the same as the general population. Monitoring of micronutrients is discussed later (Section 5.4.3).

*Recommendation*: micronutrient intakes should be regularly reviewed and calculated as there is risk of deficiency on a LRD. If lysine‐free protein substitutes and diet do not provide sufficient intake micronutrients should be supplemented. For children and adolescents on restricted natural protein intake only (see statement 17) and no protein substitute a micronutrient supplement is recommended.

### 5.2 Arginine supplementation

*The PDE consortium consensus guidelines 2021 state* ^4^:

- Arginine is part of LRT and can be given in combination with pyridoxine and a LRD (triple therapy) or with pyridoxine alone (double therapy) (statement 10, level of evidence: C).
- For newborns and infants, arginine supplementation should be started at a dose of 200 mg/kg/day whether arginine is provided alone or in combination with a LRD (statement 14, level of evidence: D).
- In children and adolescents, no consensus was reached on the dose (statement 18).
- In adults, arginine supplementation should be started at 4 g/m^2^/day with a maximum dose of 5.5 g/m^2^/day (statement 19, level of evidence: D).

*Questionnaire results*: arginine is prescribed as a single supplement by 65% (*n* = 26/40) respondents (Table S26—Q36). It is given as a medicine by 69% (*n* = 20/29), others give it with food (*n* = 7/29; 24%) or as a lysine‐free protein substitute (*n* = 5/29; 17%) (Table S28—Q38*). No information was requested about timings of arginine administration. The frequency varies from once to six times daily, but most prescribe three times (*n* = 14/29; 48%) or twice (*n* = 11/29; 38%) (Table S27—Q37*).

*Discussion and clinical practice experience*: arginine is a semi‐essential amino acid. In pharmacological doses, it can act as competitive inhibitor of lysine for intestinal absorption and transport across the blood brain barrier.^25^, ^27^, ^30^ Schmidt et al. showed in five healthy men that increasing doses of l‐arginine (up to 600 mg/kg/day) caused a linear decrease in whole‐body lysine oxidation. With high doses (400–600 mg/kg/day), plasma arginine increased and plasma lysine decreased below normal range.^60^ The PDE consortium guidelines states arginine is part of LRT and can be given in combination with pyridoxine (double therapy) and a LRD (triple therapy). The evidence to clearly differentiate the exact effect of arginine and a LRD separately and combined is not yet available. However, in one cross‐over study, six patients treated with triple therapy had significantly lower PDE‐specific cerebrospinal fluid, plasma and urine biomarkers and subjective improvement in development compared to treatment with pyridoxine and a LRD.^28^ In contrast, the GA1 guidelines do not recommend an additional arginine supplementation as there is no evidence for clinical benefit in GA1.^44^

As arginine competes with lysine for intestinal absorption, it could be argued that arginine is best taken with a meal. There is no evidence for timing or daily frequency of arginine administration. If LRD is too difficult, arginine supplementation alone might be more feasible.

A source of arginine will also be provided by the lysine‐free protein substitute. Arginine provided by diet and protein substitute does not routinely form part of the prescribed l‐arginine medicine dose.

In our experience, infants and young children on double therapy of pyridoxine and LRD often have plasma lysine concentrations below the recommended lower quartile of the normal reference range for age.^23^ Theoretically, supplementation with l‐arginine could lower plasma lysine levels further with risk of deficiency and this needs to be considered.

### 5.3 Practical dietetic management of lysine‐restricted diet

#### 5.3.1 Teaching patients and/or caregivers to count (protein or lysine + protein source)

*Questionnaire results*: patients and parents are mainly taught to count protein (*n* = 31/38; 82%), some do not count (*n* = 9/38; 24%). Differences occur in counting some fruit and vegetables (Table S14—Q24*).

Inclusion of animal protein food was advised by 24% (*n* = 9/38), only sometimes in 37% (*n* = 14/38) and never by 34% (*n* = 13/38) of respondents (Table S15—Q25). A variety of animal protein food are used (Table S16—Q26*). Most (*n* = 12/23; 52%) do not aim for a specified amount of animal protein each day when calculating the diet (Table S17—Q27). Some respondents teach all patients/caregivers (*n* = 5/23; 22%) or some patients/caregivers (*n* = 5/23; 22%) to aim for a certain amount of animal protein each day (Table S18—Q28).

*Discussion and clinical practice experience*: respondents used different systems for calculating and counting the LRD. The exact strategy may depend on several factors including the capacity of the family and age of treatment initiation. Patients and/or caregivers can be taught to count in grams of protein, to work with protein or lysine exchanges or with food groups (i.e., to avoid consuming a high amount of animal products). Some centres teach how to distinguish HBV protein foods which are high in lysine from LBV protein foods which are low in lysine. These groups can be exchanged (i.e., 1 g protein; ~70 to 80 mg lysine) from HBV foods such as milk, meat can be exchanged for 2 g of protein (~40 mg/g protein) from LBV foods such as cereal/vegetable foods (Table 2). As lysine is the limiting amino acid in cereal‐based foods, a diet based mainly on LBV protein foods with a high proportion of cereal foods could result in deficiency and therefore it is recommended to include some HBV protein foods. The group suggest the daily lysine intake is calculated to provide around 70% as LBV and 30% HBV protein foods, which will provide at least the safe level of protein intake for age (Table 2).^43^ For some, providing 50% as HBV and 50% as LBV protein foods may be better and will still provide around the safe level of protein intake.^43^ For families, this can be simplified to a daily protein intake and counting protein exchanges rather than lysine. One approach is to use average estimate values for LBV foods of 40 mg lysine per gram of protein^23^ and HBV dairy foods 70 mg lysine per gram of protein (Table 2) and to give a certain number of protein exchanges from each group. Different types of HBV foods could be included depending on personal preferences, but calculating more lysine per gram of protein. For manufactured foods, the most predominant ingredient(s) can be used to decide whether to count as a LBV or HBV protein food. A practical example of a child's diet is given in Supplement S2.

*Recommendation*: the LRD should be calculated based on lysine intake. Parents/caregivers should be taught to count protein or lysine and advised to give as a combination of LBV and HBV protein foods.

#### 5.3.2 Breastfeeding

*Questionnaire results*: 68% of the respondents would allow a limited amount of breastfeeding (*n* = 26/38), 25% plan to allow breastfeeding in the next newborn (*n* = 9/38) and breastfeeding would be allowed by all. (Table S7—Q17*). Most advised giving a lysine‐free protein substitute first, followed by breast feed on demand (*n* = 14/36; 39%) or alternating breast and lysine‐free feeds (*n* = 12/36; 33%) (Table S10—Q20*).

*Discussion and clinical practice experience*: breastfeeding in general has many health benefits and is encouraged by the PDE consortium.^4^, ^23^ The average lysine content in breast milk after the neonatal period is 69 mg lysine/g of protein (90 mg/100 mL),^43^ which is considerably lower than the lysine content of standard infant formula.

There is no published experience with breastfeeding in PDE patients on LRT. Partial breastfeeding (or expressed breast milk) is feasible and based on the survey results different methods exist to limit volume and thereby lysine intake (Table S10—Q20*). It is unclear if one of these methods would result in a difference in metabolic control, lysine absorption or growth. When giving a measured amount of lysine‐free formula first, the appetite of the infant and so the intake of breastmilk is reduced. With this technique, the infant receives natural protein at each feed. Giving a fixed amount of lysine‐free formula or alternating these feeds with breastfeeding could be more practical for mothers.

The daily volume of breast feeds or expressed breast milk is limited to provide the recommended lysine intake mg/kg/day (Table 2), this can be calculated using the lysine content of breast milk. A practical example of calculated breastfeeding plan is given in Supplement S2.

It is recommended to weigh the baby regularly (every 1–2 weeks initially) to adjust feeds for increasing weight and to provide the lysine requirements, which change with age. In children <3 years of age, plasma amino acids should be monitored every 3 months (see Section 5.4.1) to ensure lysine is maintained within the lower quartile of the normal reference range for age. More frequent monitoring is recommended if lysine levels are low. This paragraph also applies to bottle‐fed babies (Section 5.3.3).

*Recommendation*: breastfeeding is encouraged. Regular feed adjustments based on close monitoring of growth and plasma amino acids are essential.

#### 5.3.3 Bottle feeding

*Questionnaire results*: respondents most commonly advised mixing the standard infant formula and lysine‐free infant formula together (*n* = 17/36; 47%) or at each feed giving a measured amount of standard infant formula first followed by lysine‐free infant formula (*n* = 8/36; 22%) (Table S10—Q20*).

*Discussion and clinical practice experience*: the average lysine content of standard infant formula is 114 mg/100 mL, which is higher than breast milk (see Section 5.3.2). Different practical methods to combine standard infant formula with lysine‐free infant formula exist (Table S10—Q20*).

The daily volume of standard infant formula is limited to provide the recommended lysine intake mg/kg/day (Table 2), this can be calculated using the lysine content of the formula. The daily volume should ideally be divided evenly over 24 h. A practical example of calculated bottle‐feeding plan is given in Supplement S2. For weight monitoring, plasma amino acids measurements and feed adjustment recommendations, see Section 5.3.2.

*Recommendation*: standard infant formula (to provide lysine intake) should be divided evenly over 24 h and given with a lysine‐free protein substitute. Regular feed adjustments based on close monitoring of growth and plasma amino acids are essential.

#### 5.3.4 Introduction of lysine‐free protein substitute at an older age

*The PDE consortium consensus guidelines 2021 state* ^4^:

- In children and adolescents, a LRD may include a lysine‐free protein substitute. If the lysine‐free protein substitute is not well tolerated lysine reduction may be achieved by reducing total natural protein to the low end of age‐appropriate needs (statement 17, level of evidence: D).

*Questionnaire results*: almost half the respondents (*n* = 18/38; 47%) initiated a lysine‐free protein substitute in some or all their patients aged 1–6 years, 34% (*n* = 13/38) in patients aged 6–12 years, 11% (*n* = 4/38) in patients aged 12–18 years and 3% (*n* = 1/38) in patients >18 years (Table S3—Q12). Ten of 117 patients discontinued diet due to issues with lysine‐restriction and lysine‐free protein substitute (Tables S1 and S4—Q14*). Roughly half of the respondents (*n* = 19/34; 56%) found introduction of a lysine‐free protein substitute in children >1 year of age and teenagers more difficult, mainly due to taste aversion (Table S13—Q23). The introduction of the lysine‐free protein substitute in children >1 year of age varied, 22% (*n* = 8/36) started with the full dose, 17% (*n* = 6/36) with a small dose and increased as accepted and 47% (*n* = 17/36) differed by case (Table S11—Q21*).

*Discussion and clinical practice experience*: there is limited experience in introducing the LRD in older patients. Interestingly, only 10 patients were reported to discontinue the diet. Introducing a LRD with lysine‐free protein substitute in older children who are used to a normal diet can be challenging. For some, it can be helpful to start with a low dose of lysine‐free protein substitute once per day and continue to offer to improve taste acceptance and then increase to tolerance, this may take days or weeks. Lysine‐free protein substitutes should be given with main meals. Starting with the evening meal might be the most helpful when parents/carers have more time. It may be best to introduce the lunchtime dose last in school children. It can be helpful to establish a routine and is important to be patient.^58^ Lysine‐free protein substitute products are available in different formats from nutritional manufacturers but variety is very limited. Alternative protein substitutes may need to be tried to achieve acceptance with an older child. Palatability may be improved by adding different concentrated flavourings such as fruit juice, commercially available flavour concentrates, low protein milk substitutes and normal or low protein yoghurt. Ideally the protein substitute should not be mixed with food as this will alter its taste, be an increased quantity and so less easily consumed.

#### 5.3.5 Use of special low protein foods

*Questionnaire results*: special low protein foods such as pasta, bread, rice and biscuits were used by almost half of respondents (*n* = 18/38; 47%) while 45% (*n* = 17/38) reported not using them (Table S20—Q30*). About one‐third also used a low‐protein milk (*n* = 13/38; 34%).

*Discussion and clinical practice experience*: special low protein food and milk can be used to supplement the LRD to provide satiety, variety and energy. Not all centres recommend these, the questionnaire did not ask why but this is likely due to adequate food and energy intake from the diet, availability, cost and dislike of taste.

#### 5.3.6 Tryptophan

*Questionnaire results*: plasma tryptophan was monitored by 66% (*n* = 25/38) of respondents (Table S24—Q34). Few (*n* = 6/38; 16%) add tryptophan as an additional single supplement to ensure adequate intake (Table S21—Q31*). Reported dosages were 15–20 mg/kg/day (Table S21—Q31*).

*Discussion and clinical practice experience*: tryptophan is an essential amino acid and a precursor of two important metabolic pathways, serotonin synthesis and kynurenine synthesis.^61^ Unlike lysine, tryptophan is not catabolised via α‐AASA dehydrogenase, so normal dietary requirements can be given. However, there is risk of deficiency due to the LRD and common use of GA1 lysine‐free protein substitutes, which are very low in tryptophan, ranging from 4 to 8 mg/g of protein equivalent. Non‐symptomatic mild serotonin deficiency (low 5‐hydroxyindoleacetic acid) has been reported in a PDE patient on a lysine‐free, low tryptophan amino acid supplement.^26^, ^27^ Analysis of plasma tryptophan has always been challenging due to oxidative degradation of tryptophan.^62^ Depending on how the sample was collected and time stored for, this might lower the tryptophan concentration.

The LRD and protein substitute need to provide the normal tryptophan requirement.^43^ The tryptophan intake should be estimated if there is risk of deficiency or low plasma levels. It is recognised that many countries food databases lack precise or recent analysis on the tryptophan content of food making it very difficult to accurately assess. More PDE‐specific protein substitutes which contain tryptophan may become available in the future and reduce risk of deficiency.

*Recommendation*: tryptophan intake may be low and should be monitored particularly if protein substitute is tryptophan low or free.

#### 5.3.7 Adulthood and pregnancy

Currently, there is very limited information about adults with PDE. However, the PDE consortium guidelines 2021 do recommend initiating LRT in adults with cognitive delay, behavioural difficulties or poor seizure control.^4^ A LRD may be difficult to introduce in adult years, potential benefits and disadvantages should be discussed case by case. Arginine would be easier to initiate than diet. The individual or combined effects of LRT are currently unclear.

There are reports of women with PDE‐ALDH7A1, who had children^18^ but none of women on LRT.

### 5.4 Biochemical treatment monitoring

#### 5.4.1 Plasma amino acids

*The PDE consortium consensus guidelines 2021 state* ^4^:

- All patients treated with a LRD should have plasma amino acids measured at least every 3 months (<3 years of age) to 6 months (>3 years of age) (statement 24, level of evidence: D).
- Plasma lysine should remain in the lower quartile of the normal reference range for age.^23^

The 2014 guidelines, for infants <1 years of age, recommend plasma samples be taken at least 3 h after a meal, for children >1 year of age, to be taken at least 4 h after a meal.^23^

*Questionnaire results*: plasma lysine was the amino acid monitored by most respondents (*n* = 32/38; 84%) then tryptophan (*n* = 26/38; 68%) and other amino acids (*n* = 25/38; 66%) (Table S22—Q32*). Most aim for plasma lysine for age in: lower reference range (*n* = 22/38; 58%) and 21% (*n* = 8/38) normal reference range (Table S23—Q33).

Ninety percent (*n* = 34/38) sometimes adjust the amount of lysine/protein in the diet based on specific biochemical results (Table S25—Q35).

*Discussion and clinical practice experience*: most centres monitor plasma amino acids and adjust the diet based on these results. Plasma lysine does not accumulate in plasma. On the institution of a LRD, plasma lysine generally falls to the lower quartile of normal reference range for age. If plasma lysine is low and below the normal reference range, an increase in natural protein/lysine should be considered, considering the timing of blood sampling, consumption of natural protein or lysine‐free protein substitute, growth, if post illness as all can influence lysine concentrations. A higher level may occur due to the blood sample being taken immediately after a meal or non‐adherence to the lysine restriction. Assessing the other essential amino acids is important for addressing potential deficiencies, especially in patients on a LRD without a lysine‐free protein substitute.

The PDE consortium 2021 recommend monitoring every 3 months in <3‐year‐olds but this could be excessive if the diet is well established in >1 to 3 years old and if there are no concerns. If the diet is initiated at an older age, then more frequent monitoring may be appropriate initially.

*Recommendation*: plasma lysine should be maintained in the lower quartile of the normal reference range. Plasma amino acids should be monitored regularly and frequency based on age, if on protein substitute and adherence to diet.

#### 5.4.2 Other PDE‐specific biomarkers

*The PDE consortium consensus guidelines 2021 state* ^4^:

- All patients on LRT should have plasma and urine biomarkers Δ^1^‐P6C and/or α‐AASA measured every 6–12 months to assess treatment efficacy (statement 25, level of evidence: D).

*Questionnaire results*: plasma α‐AASA was measured by 26% (*n* = 10/38), urine α‐AASA by 21% (*n* = 8/38) and 8% measured both (*n* = 3/38). Plasma Δ^1^‐P6C was measured by 21% (*n* = 8/38), 8% (*n* = 3/38) measured urine Δ^1^‐P6C and 11% (*n* = 4/38) measured both (S1 Table S22—Q32*).

*Discussion and clinical practice experience*: plasma α‐AASA/Δ^1^‐P6C is measured by less than 26% of centres and urine even less. These are difficult to measure and analysis may not be available in all centres. Previous studies have demonstrated that LRT result in a significant decrease of α‐AASA/Δ^1^‐P6C levels compared to pre‐treatment or treatment with pyridoxine monotherapy.^21^, ^28^ Plasma/urine Δ^1^‐P6C and/or α‐AASA decrease with age but can fluctuate. Although there are anecdotal reports of patients with α‐AASA/Δ^1^‐P6C levels in the normal range, this is relatively rare. Currently, there is no known therapeutic target or ideal treatment range for α‐AASA/Δ^1^‐P6C levels. Therefore, diet is not adjusted based on Δ^1^‐P6C and/or α‐AASA results. It is however important to ensure that patients treated with LRT do not have α‐AASA/Δ^1^‐P6C levels that return to pre‐treatment values or above.

Pipecolic acid is less reliable as a biomarker and been reported to be normal in PDE patients treated with pyridoxine only.^63^, ^64^

#### 5.4.3 Micronutrients

The PDE 2014 guidelines recommend biochemical monitoring of micronutrients at 1 month and 3 months after initiation of diet then every 6 months.^23^

*Questionnaire results*: vitamins and minerals were monitored by 63% of respondents (*n* = 24/38) (Table S22—Q 32*).

*Discussion and clinical practice experience*: interestingly only 63% monitored vitamins and minerals. To ensure adequate nutritional status, biochemical measurement of micronutrients is recommended especially when patients are following a LRD with no lysine‐free protein substitute or additional supplements as they are at risk of micronutrient deficiencies. As a minimum, it is suggested to monitor iron status, vitamin B12 and vitamin D as deficiencies have been reported in other protein‐restricted diets.^23^, ^56^ Including functional parameters is recommended as they may inform about cellular physiology as well (ferritin, haemoglobin, mean corpuscular volume for iron; methylmalonic acid or total homocysteine in serum for cellular vitamin B12). The diet should also be assessed for adequacy of intake at all out‐patient reviews, at least annually.

*Recommendation*: biochemical monitoring of micronutrient status of diet is recommended.

### 5.5 Emergency treatment

*The PDE consortium guidelines 2021 state* ^4^:

- In times of seizure relapse during febrile illness, the dose of pyridoxine may be doubled up to a maximum of 60 mg/kg/day (in children) or 500 mg/day (adolescents and adults) for up to 3 days (statement 28, level of evidence D).
- In times of illness, ensure adequate caloric intake to prevent catabolism of endogenous protein and reduce protein intake (statement 29, level of evidence D).

*Questionnaire results*: only half the respondents (*n* = 20/40; 50%) prescribe an emergency regimen for some or all patients during episodes of illness and poor oral intake (Table S29— Q39). The main reason for use was to prevent catabolism (*n* = 16/20; 80%) and to prevent risk of breakthrough seizures (*n* = 13/20; 65%) (Table S30—Q40*). Pyridoxine dose was doubled by 55% (*n* = 11/20) (Table S31—Q41*). The usual daily energy intake was prescribed by 40% (*n* = 8/20) and increased by 20% (*n* = 4/20). Few decreased (*n* = 6/20; 30%) and/or stopped (*n* = 3/20; 15%) natural protein intake temporarily (Table S31—Q41*).

*Discussion and clinical practice experience*: acute metabolic decompensation during intercurrent illness is not a clinical finding but there is risk of breakthrough seizures. It can be hypothesised that during catabolism the metabolites α‐AASA, Δ^1^‐P6C and pipecolic acid could increase and potentially inactivate PLP even more but there are no data to support this.

For both reasons, it appears prudent to give guidance on illness management. Pyridoxine dose may be increased and a solution of glucose polymer given, akin to standard emergency regimens is advised.^65^ More practical advice for illness management is provided below (Table 4).

**TABLE 4:** Guidance for illness management.

Diet	Dietary advice
Pyridoxine	During febrile illness to prevent risk of seizure or to treat seizures, the dose of pyridoxine may be doubled up to a maximum of 60 mg/kg/day (in children) or 500 mg/day (adolescents and adults) for up to 3 days.^4^
Lysine‐free protein substitute	Continue to give the usual dose of lysine‐free protein substitute, if possible. It may be better to give this in smaller more frequent doses throughout the day.
High carbohydrate intake and fluids	Encourage frequent glucose polymer solution or high carbohydrate drinks to prevent catabolism. There is however no evidence to give more energy than the child's usual requirement. Suggested carbohydrate concentration of solutions for age, based on standard emergency regimens: 10% up to 1 year, 15% 1–2 years, 20% from 2 to 9 years and 25% >10 years of age.^65^ The daily fluid volume of glucose polymer/high carbohydrate drinks to be around maintenance fluid requirements for age if the child is not eating.
Natural protein intake	If on restricted‐lysine diet there is no need to ‘formally’ omit all the natural protein. In practice a reduced appetite leads to a lower natural protein intake.
Arginine	Continue to give the usual dose of arginine supplementation.
Anti‐pyretic medication	Give as necessary for high temperatures.
Intravenous supplementation	Intravenous fluids (10% glucose) and pyridoxine supplementation should be administered only if justified by the severity of the acute disease and inability to tolerate medications or fluids orally or enterally.

*Recommendation*: an illness management plan to limit catabolism and reduce risk of breakthrough seizures is recommended.

## 6 CONCLUSION AND FUTURE RECOMMENDATIONS

Although the evidence is still limited, the PDE consortium 2021 guidelines suggest LRT be started early in life for optimal neurological outcome.^4^ Even later in childhood and adolescence LRT may be useful in preventing deterioration or further damage. These dietary management recommendations include data from the first international questionnaire on dietary management in PDE patients. Results showed most centres still have limited experience (one to five patients) and treatment strategies vary, which underlines the necessity for a more standardised approach. As the evidence is still very limited, these recommendations are highly dependent on expert opinion. The group have reviewed and updated the 2014 guidelines and provide practical dietetic recommendations for LRD. There are calculated examples of LRD for infants and children and recommendations for biochemical and nutritional monitoring.

Future research is essential to increase knowledge about the individual added value of LRD and arginine supplementation and to optimise the practical application of these therapies. We acknowledge the difficulty in performing studies in a very rare disease and urge for multi‐centre studies. Studies should compare the value of LRD versus arginine supplementation. There is also a need to find biomarkers to assess the effectiveness of LRT. These dietary management recommendations should be repeated in the next 5 years. The role of lysine‐free protein substitutes need further evaluation such as doses for different age ranges and the need for these in older children on LRD. It would also be important to explore the use of emergency regimens.

## AUTHOR CONTRIBUTIONS

AMJvW, MDi, CRC and CvK initiated the study. MDi, CM, LB, MDr, SG and AMJvW designed the questionnaire. MDr and AMJvW analysed the data. MDi, CM and AMJvW interpreted the data and were the lead writers of the manuscript. LB, MDr and SG interpreted the data and co‐wrote the manuscript. CvK and CRC aided in the revision of the manuscript for content. All authors read and approved the manuscript.

## FUNDING INFORMATION

No funding was received for this study.

## CONFLICT OF INTEREST STATEMENT

Annemiek M. J. van Wegberg has received a research grant from Nutricia, travel support from Nutricia and Vitaflo and participated in nutritional product research evaluation studies for Vitaflo International Ltd UK. Marjorie Dixon has received honoraria, travel support from Danone Nutricia and Vitaflo International Ltd and participated in nutritional product research evaluation studies for Vitaflo International Ltd and Nutricia, UK. Clara D. M. van Karnebeek was the principal investigator in nutritional product research evaluation studies for Vitaflo International Ltd. Chloe Millington participated in nutritional product research evaluation studies for Vitaflo International Ltd. Sommer Gaughan received an honorarium from Nutricia North America for PDE case write up. The other authors (Laurie Bernstein, Curtis R. Coughlin II, Morgan Drumm) declare no conflict of interest.

## ETHICS STATEMENT

This article does not contain any studies with human or animal subjects performed by any of the authors.

## Supporting information


**Data S1.** Results of questionnaire.

**Data S2.** Examples and calculations.

## Data Availability

The data that supports the findings of this study are available in the supplementary material of this article.
