# Supplementary material for: Dietary management for pyridoxine‐dependent epilepsy due to α‐aminoadipic semialdehyde dehydrogenase deficiency, a follow‐on from the international consortium guidelines
Source: JIMD Rep. 2024 Apr 3;65(3):188–203. doi: 10.1002/jmd2.12418 (PMC11078710; doi:10.1002/jmd2.12418)
Supplement: Supplementary file 1 — Data S1. Results of questionnaire. [file JMD2-65-188-s002.docx]

**Supplemental File 1 – results of questionnaire**

**Supplemental Table 1 – question 1, 4 5, 7, 8, 9, 10, 13:**

|  | | **Number of PDE patients the clinic cares for** | | **Type of treatment** | | | | **Dietary treatment** | | |
| --- | --- | --- | --- | --- | --- | --- | --- | --- | --- | --- |
| **Continent** | **Country** | **0-18 years** | **>18 years** | **Pyridoxine** | **Double therapy (pyridoxine + diet)** | **Double therapy (pyridoxine + arginine)** | **Triple therapy** | **Lysine restricted diet** | **Amino acid supplement** | **Discontinued Lysine restricted diet** |
| Asia | India | 10 | - | Yes | 10 | - | - | Yes | - | No |
| Asia | Pakistan | 4 | - | Yes | - | - | 4 | Yes | 4 | No |
| Europe | Denmark | 1 | - | Yes | - | - | 1 | Yes | 1 | No |
| Europe | Estonia | 1 | - | Yes | - | - | 1 | Yes | 1 | No |
| Europe | Estonia | 1 | - | Yes | - | - | 1 | Yes | 1 | No |
| Europe | Italy | 1 | - | Yes | - | - | - | No | - | - |
| Europe | Norway | 5 | - | Yes | 1 | - | 4 | Yes | 5 | No |
| Europe | Russian Federation | 3 | - | Yes | - | - | - | No | - | - |
| Europe | Spain | 3 | - | Yes | - | - | 3 | Yes | 3 | No |
| Europe | the Netherlands | 5 | - | Some (n=3) | - |  | 3 | Yes | 5 | No |
| Europe | the Netherlands | 7 | 8 | Yes | - | 3 | 2 | Some | - | No |
| Europe | the Netherlands | 2 | - | Yes | - |  | 2 | Yes | 1 | No |
| Europe | United Kingdom | 22 | - | Yes | 13 | 4 | 1 | Some | 14 | Yes (n=4) |
| Europe | United Kingdom | 2 | - | Yes | 2 | - | - | Yes | 2 | No |
| Europe | United Kingdom | 2 | - | Yes | 1 | - | 1 | Yes | 2 | No |
| Europe | United Kingdom | 10 | - | Yes | 2 | 1 |  | Some | 2 | No |
| Europe | United Kingdom | 4 | - | Yes | - | 1 | 3 | Some | 3 | Yes (n=1) |
| North America | Canada | 1 | - | Yes | 1 | - | - | Yes | 1 | No |
| North America | Canada | 3 | - | Yes | - | - | 3 | Yes | 3 | No |
| North America | Canada | 3 | - | Yes | - | 1 | 2 | Some | 2 | No |
| North America | Canada | 10 | - | Yes | - | 3 | 7 | Some | 7 | Yes (n=1) |
| North America | Canada | 8 | - | Yes | - | 3 | 5 | Some | 5 | Yes (n=1) |
| North America | United States | 2 | - | Yes | - | - | 2 | Yes | 1 | No |
| North America | United States | 1 | - | Yes | - | - | 1 | Yes | 1 | No |
| North America | United States | 4 | - | Yes | - | - | 4 | Yes | 2 | No |
| North America | United States | 4 | - | Yes | - | 1 | 3 | Yes | 3 | Yes (n=1) |
| North America | United States | 4 | - | Yes | - | 2 | 2 | Some | - | Yes (n=2) |
| North America | United States | 1 | - | Yes | - | - | 1 | Yes | 1 | No |
| North America | United States | 2 | - | Yes | - | 1 | 1 | Some | 1 | No |
| North America | United States | 1 | - | Yes | - | - | 1 | Yes | 1 | No |
| North America | United States | 1 | - | Yes | - | - | 1 | Yes | 1 | No |
| North America | United States | 2 | 1 | Yes | - | 1 | 2 | Some | 2 | Yes (unable to tolerate medical food) |
| North America | United States | 1 | - | Yes | - | - | 1 | Yes | 1 | No |
| North America | United States | 1 | - | Yes | - | - | 1 | Yes | 1 | No |
| North America | United States | 2 | - | Yes | - | - | 2 | Yes | 1 | No |
| North America | United States | 4 | - | Yes | - | - | 4 | Yes | 4 | No |
| North America | United States | 2 | - | Yes | 1 | - | 1 | Yes | 2 | No |
| Oceania | Australia | 2 | - | Yes | 1 | - | 1 | Yes | 2 | No |
| Oceania | Australia | 1 | - | Yes | - | - | 1 | Yes | - | No |
| Oceania | Australia | 3 | - | Yes | 1 | - | - | Some | 1 | No |
| **TOTAL** |  | **146** | **9** | **153** | **33** | **21** | **72** |  | **87** |  |

**Supplemental Table 2 – question 11**In infants under 12 months of age, what treatment do you ROUTINELY give in addition to pyridoxine once diagnosis of PDE is confirmed? *Note: multiple answers are possible.*
**Total n = 38**

| Pyridoxine + lysine restricted diet | Pyridoxine + lysine restricted diet + lysine free protein substitute | Pyridoxine + lysine restricted diet + arginine | Pyridoxine + lysine restricted diet + lysine free protein substitute + arginine | Other |
| --- | --- | --- | --- | --- |
| 2 (5,3%) | 9 (23,7%) | 3 (7,9%) | 23 (60,5%) | 3 (7,9%) |

*Specification of other:
Have not seen an infant.
I have not treated any PDE infants
We recommend triple therapy. Not all families choose to follow diet restriction.*

**Supplemental Table 3 – question 12**
Have you initiated a lysine restricted diet (with or without lysine-free protein substitute) in older children who have been on pyridoxine only since diagnosis? *Please comment on each age group.*
**Total n = 38**
I aim for..

|  | LRD and PS in all patients | LRD and PS in some patients | LRD only in all patients | LRD only in some patients | No patients in this age group | None of these | Not responded |
| --- | --- | --- | --- | --- | --- | --- | --- |
| Age 1-6 years | 16 (42,1%) | 2 (5,3%) | 1 (2,6%) | 3 (7,9%) | 7 (18,4%) | 6 (15,8%) | 3 (7,9%) |
| Age 6-12 years | 12 (31,6%) | 1 (2,6%) | 0 | 1 (2,6%) | 11 (28,9%) | 7 (18,4%) | 6 (15,8%) |
| Age 12-18 years | 3 (7,9%) | 1 (2,6%) | 0 | 3 (7,9%) | 17 (44,7%) | 7 (18,4%) | 7 (18,4%) |
| Age >18 years | 1 (2,6%) | 0 | 0 | 0 | 18 (47,4%) | 7 (18,4%) | 12 (31,6%) |

*LRD: lysine restricted diet. PS: lysine-free protein substitute*

*Comments made in this question:*He was treated with diet since 2 month old
?
Two patients aged 9 year are on a diet. A protein substitute was aimed for, but not achieved. Five patients aged 13, 16, 16, 17, 17 are not on a diet. The diet was discussed in most, but eventually not initiated. I however aim for at least diet only in some patients.
the older child came to us on the diet already
In older children diet is attempted. First to see if pts tolerate substitute & if so then full diet is initiated.
Not applicable
n=1

**Supplemental Table 4 – question 14**
Why have your patients discontinued lysine restricted diet? *Note: multiple answers are possible.*
This question was answered by **7 respondents.**

| **Answer option** | **Frequency** | **Percent** |
| --- | --- | --- |
| The family perceived no clinical improvement | 3 | 42,9 |
| Not possible to even initiate lysine restricted diet | 1 | 14,3 |
| Unable to adhere to the prescribed protein/lysine restriction | 6 | 85,7 |
| Risk of nutritional deficiencies | 0 | 0 |
| Unable to take the prescribed dose of lysine free protein substitute | 3 | 42,9 |
| Not able to tolerate the taste of lysine-free protein substitutes | 3 | 42,9 |
| Diet not feasible, due to patients circumstances (e.g. behavioral issues, understanding) | 3 | 42,9 |
| Diet not feasible, due to family circumstances (e.g. caregivers could not sustain the diet or found it too challenging) | 3 | 42,9 |
| Other (please specify) | 1 | 14,3 |

Specification other: reason unclear

**Supplemental Table 5 – question 15**Which recommendations do you use to devise the diet plan for PDE patients? *Note: please answer all components (multiple answers are possible for each)***Total n = 38**

|  | PDE consortium guidelines 2014 | GA 1 guidelines 2017 | Local/country recommendations | FAO/WHO/UNU 2007 | None of these |  |
| --- | --- | --- | --- | --- | --- | --- |
| Lysine mg/kg/day | 30 (78,9%) | 9 (23,7%) | 1 (2,6%) | 2 (5,3%) | 4 (10,5%) |  |
| Lysine free protein substitute g/kg/day | 27 (71,1%) | 14 (36,8%) | 4 (10,5%) | 0 | 6 (15,8%) |  |
| Total protein intake g/kg/day | 29 (76,3%) | 14 (36,8%) | 10 (26,3%) | 5 (13,2%) | 1 (2,6%) |  |
| Energy intake (kcals/kg/day) | 21 (55,3%) | 10 (26,3%) | 10 (26,3%) | 11 (28,9%) | 3 (7,9%) |  |

*Comments made in this question:*Difficult communication so we can only count protein else we would have counted lysin. But he is recommend no meet, chicken or any type of cheese
whatever it takes to ensure good wt gain
Energy based on needs (wt gain/wt loss, etc)

**Supplemental Table 6 – question 16**How do you calculate the daily protein/lysine intake to prescribe a patient? *Select the answer that applies the most.*
**Total n = 38**

| **Answer option** | **Frequency** | **Percent** |
| --- | --- | --- |
| Calculate total grams of protein/day | 14 | 36,8 |
| Calculate both mg of lysine/day and grams of protein/day | 8 |  |
| Calculate total grams of protein/day, then estimate the lysine this provides. | 7 | 18,4 |
| Calculate total mg lysine/day then calculate the protein needed to provide this. | 4 | 10,5 |
| Calculate total mg of lysine/day | 1 |  |
| Other (please specify) | 4 |  |
|  |  |  |

Specifications of other:
Calculate total grams of protein/day, distinguish high and low lysine products (kölker)
calculate lysine, but have families count protein
Initially started at 4 months of age with counting mg lysine, now tolerating vegetarian diet
Estimate current protein and lysine intake and compare to blood lysine levels aiming to keep lysine in the lower reference range for age

**Supplemental Table 7 – question 17**Do you allow infants on lysine restricted diet to breast feed? *Note: multiple answers are possible.*
**Total n = 38**

| **Answer option** | **Frequency (%)** | |
| --- | --- | --- |
| Yes, a limited amount of breast feeding is allowed | 20 (52,6%) | |
| Yes, a limited amount of expressed breast milk fed from the bottle is allowed | 6 (15,8%) |  |
| Not yet, we plan to allow breast feeding in the next newborn with PDE | 9 (23,7%) | |
| No | 0 | |
| Other (please specify) | 7 (18,4%) | |

Specifications of other:
This has not been an option for us yet as the infant we had was already bottle feeding standard infant formula. I would consider it with the next infant diagnosis.
Our patients were diagnosed after infancy
Only had a formula-fed child. Would not know for breastfeeding
have not started an infant, but would allow limited breast feeding
no experience with infants so far
ad lib and use estimate of protein and lysine intake and labs to guide restriction
I have not encountered this situation.

**Supplemental Table 8 – question 18**Do you prescribe a lysine-free protein substitute for your patients?
**Total n = 38**

|  | **Frequency** | **Percent** |
| --- | --- | --- |
| Yes | 32 | 84,2 |
| No | 2 | 5,3 |
| Some | 4 | 10,5 |

**Supplemental Table 9 – question 19**
Which type of lysine-free protein substitute(s) do you use? *Note: multiple answers are possible.*
**Total n = 36**

|  | **Frequency** | **Percent** |
| --- | --- | --- |
| GA1 specific protein substitute | 35 | 97,2 |
| PDE specific protein substitute | 1 | 2,8 |
| Other (please specify) | 0 | 0 |

*Note: the one who answered PDE specific also answered GA1.*

**Supplemental Table 10 – question 20**
How do you give the lysine-free protein substitute to infants <12 months of age? *Note: multiple answers are possible.***Total n = 36**

| **Answer option** | **Frequency (%)** |
| --- | --- |
| At each feed the lysine-free protein substitute is mixed together with standard infant formula | 17 (47,2%) |
| At each feed a measured amount of standard infant formula is given first then followed by lysine-free protein substitute | 8 (22,2%) |
| At each feed a measured amount of lysine- free protein substitute is given first followed by standard infant formula on demand | 6 (16,7%) |
| At each feed a measured amount of expressed breast milk is given first then followed by lysine-free protein substitute on demand | 3 (78,3%) |
| At each feed a measured amount of lysine-free protein substitute is given first then followed by a breastfeed on demand | 14 (38,9%) |
| At each feed a measured amount of the lysine-free protein substitute is given first followed by a measured amount of expressed breast milk | 4 (11,1%) |
| At each feed either a lysine-free protein substitute or a breast feed/ expressed breast milk is given (alternating) | 12 (33,3%) |
| Other (please specify | 8 (22,2%) |

Specifications of other:
Evt. A fixed number of protein substitute meas followed by free number of breast feeds or a fixed number of infant formula meals and free amount of proten substitute meals.
Wish of parents is important.
we didn't give the lysine-free protein subsitute in young children <12 montths
Our patients were diagnosed after infancy
Set amount lysine free protein substitute split into doses over the day and breast feeds on demand
no infants yet
I have not encountered this scenario.
A measured amount of lysine free formula is given pre breast milk or infant formula at a number of feeds per day (up to 5 feeds during the day and not at night) but not all feeds

**Supplemental Table 11 – question 21**
How do you introduce the lysine-free protein substitute(s) in children >1 years of age and teenagers? *Note: multiple answers are possible.*
**Total n = 36**

| **Answer option** | **Frequency (%)** |
| --- | --- |
| 1/4 of the daily requirement and increase as accepted | 3 (8,3%) |
| 1/3 of the daily requirement and increase as accepted | 2 (5,6%) |
| 1/2 of the daily requirement and increase as accepted | 1 (2,8%) |
| Full dose and evaluate how it is accepted | 8 (22,2%) |
| I use a specific stepwise introduction program | 5 (13,9%) |
| This differs per case | 17 (47,2%) |
| I do not prescribe lysine free protein substitute to older patients | 2 (5,6%) |
| Other (please specify) | 7 (19,4%) |

Specification of other:
I have not had at patient > 1 year
the patient has been initiated in an other hospital, but in generally we decide that per case (differs what the natural protein toleration is and IF / how much you have to supplement with the lysine free protein substitute
Usually offer different products according to what they may be likely to mix it in to increase chance of accepting the product
no patients fit this criteria
Have not had experience with this yet
Not done yet
N/A have not had to introduce to any pt > 1

**Supplemental Table 12 – question 22**
How often do you advise the lysine-free protein substitute is given throughout the day in older children and teenagers with PDE? *Note: multiple answers are possible.*
**Total n = 34**

| **Answer option** | **Frequency (%)** |
| --- | --- |
| Once per day | 2 (5,9%) |
| Twice per day | 11 (32,4%)) |
| Three times per day | 21 (61,8%) |
| Four times per day | 4 (11,8%) |
| Other (please specify) | 9 (26,5%) |

Specification other:
our patient is 2 years
At each main meal, 4-5 meals
ideally 3 times a day if they can
we have none
Would say 2-3 times
N/A only pt is <2 years old
No children this age
DO NOT HAVE OLDER CHILDREN WITH PDE
our child is not on the Gel as getting enough protein from vegetarian diet

*Note: 7 answered: 2-3 times; 3 answered 3-4 times; 1 answered 1,2,3 times.*

**Supplemental Table 13 – question 23**
Have you found it more difficult to initiate a lysine-free protein substitute in children >1 year of age and teenagers, who started diet later? *Select the answer that applies the most.***Total n = 34**

| **Answer option** | **Frequency (%)** |
| --- | --- |
| No | 9 (26,5%) |
| Yes | 14 (41,2%) |
| Some | 5 (14,7%) |
| Not answered | 6 (17,6%) |
|  |  |

Please explain the reasons why it has been difficult and give the age of child(ren) if possible
Taste aversion
I have not tried
No patients at this age
we have only one patient
Easy due to gastrostomy
We tried in our 2 patients on diet, they both did not accept the taste in the end
I don't have much experience with this group of patients
taste, frequency of giving, socially difficult for teenagers
taste
The children with PDE seem to accept the lysine free protein substitute more easily when started at a later age than children with other conditions have.
we have none
N/A
Not specific experience with lysine-free protein substitute, but with other disorders it is a challenge to introduce the new taste and texture of these substitutes.
Have not had this experience yet
Taste of formula difficult in older children and teenagers
aversion to taste
Taste/acceptance
Age 6, taste acceptance. Now accepts mixed with juice.

*Note: This was also answered by people who answered no. The blue lines represent people who answered yes, the orange one who answered some.*

**Supplemental Table 14 – question 24**Do you teach patients and/or caregivers to count in lysine (mg) or in protein (g) or a combination? *Note: multiple answers are possible.*
**Total n = 38**

| **Answer option** | **Frequency** | **Percent** |
| --- | --- | --- |
| Count lysine (mg) intake from all foods | 1 | 2,6% |
| Count lysine (mg) intake for most foods except fruits and some vegetables | 1 | 2,6% |
| Count protein (g) intake from all foods | 13 | 34,2% |
| Count protein (g) intake for most foods, except fruits and some vegetables | 18 | 47,4% |
| Count in a combination of lysine (mg) and protein (g) for all foods | 0 | 0 |
| Count in a combination of lysine (mg) and protein (g) for most foods, except fruits and some vegetables | 3 | 7,9% |
| No counting system, patients are instructed without the need to count lysine or protein | 9 | 23,7% |
| Counting system varies based on age | 3 | 7,9% |
| Other, please specify | 6 | 15,8% |

| Please explain your answer with a description of how you teach the diet:   - Exact foods are mentioned in plan with required amounts in front of them , food substitutes are given in a separate list which have same exchanges, list of all foods are given from which they can chhise and replace the given food. - Req is 18 g of protein. *Try to keep the amount of dairy and meat/fish (High lysine food) stable from day to day. - distinguish between high and low lysine products. - Parents are instructed to count/estimate in grams protein. The foods are divided in high lysine containing foods and low lysine containing foods. These can be exchanged. 0,5 gram of protein high-lysine food can be exchanged for 1,0 gram of protein low-lysine food. Some families just estimate the protein intake without this exchange system. - as above example - Lysine 35-40 mg/kg x 42.5 = 1700mg/day, Dietary protein is divided into 70% from vegetables and cereal and 30% form dairy, 70% of 1700mg = 1190mg/40mg = 30g protein from vegetables and cereal, 30% of 1700mg = 510mg/70mg = 7g from Dairy Total from lysine foods exchanges = 37g - It is dependant on the family and what they are able to understand. It may be simple advise regarding reducing intake of high protein foods. Other wise we would teach to count in grams of protein. - depending on family intellect and coping - Estimated 1g of protein = 50mg lysine - Some high Lys fruits are counted (ex: watermelon) - We use labels to count protein. - Track intake in grams of protein (if family is capable of counting protein); if family cannot count protein, use a "food patterns" approach (e.g., one 2 oz serving of meat per week, 4 oz dairy per day, etc.) - higher lysine foods/ items will be given an exchange to count grams of protein at a higher amount than they actually provide - I advise a primarily vegetarian diet and how to count mg of lysine from non-animal protein foods. - We use specific grams of protein for food. We do not round. If a food is 0.5 g protein we count it as 0.5 not 1.0 - taught to limit to 1-2 protein sources in appropriate servings, restrict high protein foods that would exceed limit (animal proteins), or count grams of protein and provide protein gram goal per day. - Carer is taught grams of protein from grains, some veg/fruit. Alternatively for older commencing child, reducing portions/swapping out higher protein foods was recommended. Some other patients are taught to count '5g protein portions' from higher protein foods (but not needing to count fruit/veg/grains). Depends on age, clinical indications/severity, and ability/literacy of family. - patient is tolerating vegetarian diet - Depending on the patient will either just work in food groups or else count all protein |
| --- |

**Supplemental Table 15 – question 25**Do you advise animal protein foods are included in the diet? *Select the answer that applies the most.***Total n = 38**

|  | Frequency | Percent |
| --- | --- | --- |
| Yes | 9 | 23,7 |
| No | 13 | 34,2 |
| Sometimes (please specify) | 14 | 36,8 |
| No answer | 2 | 5,3 |

Specification of sometimes:

- Clear soups in case intake is bad in winters in calculated amounts
- No because the parent cannot count lysine in case intake is bad in winters in calculated amounts
- Some dairy
- All the parents I have now like to use some animal protein foods, I don´t say they have to
- Depends on the amount of protein a patient can have. We advise for example a small amount of yoghurt.
- very carefully measured and limited
- For older children, dairy products may be included to meet Lys needs
- depends on protein allowance and patient likes/dislikes
- If on medical food, may not need animal protein in the diet
- Milk
- No, but the family of the one patient we have is vegetarian this has not been explored.
- Sometimes a bit of dairy, peanut butter is allowed if unable to take medical food and or meet protein needs
- only rarely
- Dairy occasionally

**Supplemental Table 16 – question 26**Which animal protein foods do you advise are included in the diet? *Note: multiple answers are possible.***Total n = 23**

| **Answer option** | **Frequency** | **Percent** |
| --- | --- | --- |
| Milk, yogurt, fromage frais | 14 | 60,9 |
| Cheese | 8 | 34,8 |
| Egg | 5 | 21,7 |
| Meat/Poultry | 3 | 13,0 |
| Fish | 3 | 13,0 |
| This varies per case based on dietary habits | 17 | 73,9 |
| None | 2 | 8,7 |
| Other (please specify) | 7 | 18,4 |

Specification of other:
- Clear soups
- Dairy but needs be counted in protein goal.
- depending on family habits and preferences
- mostly limited/restricted but can include if protein goal allows
- peanut butter
- Some of the older children have egg, fish, eat if on higher protein intake. Others have milk allergic to dairy so has meat, fish.
- We ask the families to provide a mix of higher & lower lysine containing foods - the animal proteins are a higher source of lysine

**Supplemental Table 17 – question 27**What proportion of daily protein/lysine intake do you aim to provide from animal protein food? ﻿*Select the answer that applies the most.*
**Total n = 23**

|  | Frequency | Percent |
| --- | --- | --- |
| 1/3 | 3 | 13,0 |
| 1/2 | 2 | 8,7 |
| I do not aim for a certain proportion | 12 | 52,2 |
| Other (please specify) | 5 | 21,7 |
| No answer | 1 | 4,3 |

Specification of other:
<25%
Most important to try to keep it stable from day to day, most give 1/3 to ½ of animal protein
A specific serving size to provide the lysine required (ex: a specific amount of cows milk or yogurt)
Not a proportion, I adjust based on current intake in relation to labs
Depends on the patient usually at least 1/3

**Supplemental Table 18 – question 28**Do you teach patients/caregivers to aim for a certain proportion of protein/lysine intake from animal products? *Select the answer that applies the most.*
**Total n = 23**

|  | **Frequency** | **Percent** |
| --- | --- | --- |
| Yes | 5 | 21,7 |
| No | 12 | 52,2 |
| Sometimes (please specify) | 5 | 21,7 |
| No answer | 1 | 4,3 |

Specification of sometimes:
Yes in case of poor intake but not often its all in calculated amounts
depending on the amount of natural protein a patient can take.
I do if that is the food that they prefer when we take a diet history
I would encourage animal products
If not taking medical food regularly, aim for 1-2 small servings of animal protein per day

**Supplemental Table 19 – question 29**How do you advise parents to count the protein/lysine intake initially? *Note: multiple answers are possible.***Total n = 38**

| **Answer option** | **Frequency** | **Percent** |
| --- | --- | --- |
| Weigh food | 18 | 47,4 |
| Household measures | 22 | 57,9 |
| Do not advise any counting system | 3 | 7,9 |
| Other (please specify) | 8 | 21,1 |

Specification of other:
- on intuition if possible
- I start to teach how to weigh food; when parents are used to weigh, they usually now how much they can give to their child; then sometimes it becomes household measures.
- Depends on parental ability
- only avoid certain food groups
- Nutrition Facts Table and serving sizes
- Household was recommended or ease and practicality but the 1 family we have also likes to weigh.
- weigh food if they are infants or young children. Household measurements if attempting diet in an older child/teenager
- read food labels

**Supplemental Table 20 – question 30**Do patients use special manufactured low protein foods and milk in their diet. This refers to medical foods or prescription low protein foods, not supermarket products. *Note: multiple answers are possible.***Total n = 38**

| **Answer option** | **Frequency** | **Percent** |
| --- | --- | --- |
| Yes- Low protein food (pasta, bread, rice, biscuits, etc.) | 18 | 47,4 |
| Yes- Low protein milk | 13 | 34,2 |
| No | 17 | 44,7 |
|  |  |  |

Additional comments to this question:
- sometimes gluten free with less protein
-The amount varies on the age and the appetite of the child
-They use these depending on their individual preferences
-Small amounts only as required
-Depending on requirements: my patients can tolerate regular grain products & some dairy product
-Some special low protein foods (meat and cheese alternatives)
-Almond/rice milk, no medical foods
-No currently but considering this for the future if indicated/needed.
- primarily use for animal protein substitutions, often regular grains can be used in the diet
-They have been able to maintain lysine within lower quartile without medical foods
-Patient is now 18 months and taking a small amount of GA1 Anamix infant

**Supplemental Table 21 – question 31**How do you ensure adequate tryptophan (TRP) in the diet? *Note: multiple answers are possible***Total n = 38**

| **Answer option** | **Frequency** | **Percent** |
| --- | --- | --- |
| Diet | 26 | 68,4 |
| GA1 lysine free protein substitute low in tryptophan | 20 | 52,6 |
| PDE specific lysine free protein substitute with tryptophan | 2 | 5,3 |
| As an additional single supplement (please specify mg/kg/day) | 6 | 15,8 |

Specification dose:
20 mg/kg/dag
15 mg / kg / day
Added to lysine free protein substitute formula or given as a medication
Prescribed by MD
not sure, prescribed by MD
if serum levels indicate need

**Supplemental Table 22 – question 32**Which biochemical parameters do you monitor? *Note: multiple answers are possible*.
**Total n = 38**

| **Answer option** | **Frequency** | **Percent** |
| --- | --- | --- |
| Plasma lysine | 32 | 84,2 |
| Plasma tryptophan | 26 | 68,4 |
| Other amino acids | 25 | 65,8 |
| Urine α-AASA | 11 | 28,9 |
| Plasma α-AASA | 13 | 34,2 |
| Urine Δ1-P6C | 7 | 18,4 |
| Plasma Δ1-P6C | 12 | 31,6 |
| Urea and electrolytes | 10 | 26,3 |
| CSF amino acids and neurotransmitters | 2 | 5,3 |
| Vitamins and minerals | 24 | 63,2 |
| Other (please specify) | 9 | 23,7 |

*Note: Regarding α-AASA , 26% of the respondents (n=10/38) measured plasma α-AASA, 21% respondents (n=8/38) measured urine α-AASA and 8% (n=3/38) measured both plasma and urine α-AASA. With regard to Δ1-P6C 21% of the respondents (n=8/38) measured plasma Δ1-P6C, 8% of the respondents (n=3/38) measured urine Δ1-P6C and 11% (n=4/38) respondents measured both plasma and urine Δ1-P6C*

Specification other:
none actually
serum pipecolic acid
the physician will give an advise about the diet
2-OPP
CSF AASA

The geneticist may monitor other labs

Acylcarnitine Bloodspot
not 100% sure what we monitor (urine vs plasma for AASA)
Urine amino acids, urine creatine metabolites, urine purine and pyrimidines

**Supplemental Table 23 – question 33**What plasma lysine concentration do you aim for? *Select the answer that applies the most.*
**Total n = 38**

| **Answer option** | **Frequency** | **Percent** |
| --- | --- | --- |
| Normal reference range for age (local lab parameters) | 8 | 21,1 |
| Lower reference range for age (local lab parameters) | 22 | 57,9 |
| High normal reference range | 1 | 2,6 |
| Other (please specify) | 4 | 10,5 |
| Not answered | 3 | 7,9 |

Specification other:
As per PDE guidelines
60-80umol/L (with a bare minimum of 40-50)
60-120 uM
Lower quartile of reference range

**Supplemental Table 24 – question 34**Do you monitor tryptophan? *Select the answer that applies the most.***Total n = 38**

| **Answer option** | **Frequency** | **Percent** |
| --- | --- | --- |
| Yes | 25 | 65,8 |
| No | 10 | 26,3 |
| Not answered | 5 | 13,2 |

**Supplemental Table 25 – question 35**Do you adjust the amount of lysine/protein in the diet based on specific biochemical results?
**Total n = 38**

| **Answer option** | **Frequency** | **Percent** |
| --- | --- | --- |
| Yes/Sometimes | 34 | 89,5 |
| No | 1 | 2,6 |
| Not answered | 3 | 7,9 |

**Supplemental Table 26 – question 36**Do you prescribe I-arginine as a single supplement? *Select the answer that applies the most.*

**Total n = 40**

| **Answer option** | **Frequency** | **Percent** |
| --- | --- | --- |
| Sometimes (When do you prescribe?) | 3 | 7,5 |
| Yes | 26 | 65,0 |
| No | 8 | 20,0 |
| Not answered | 3 | 7,5 |

Specification of sometimes:
If do not want or cannot be on diet.
Used in patients where diet is not tolerated
Doctor dependent

**Supplemental Table 27 – question 37**

How many times per day do you advise I-arginine supplement is given? *Note: multiple answers are possible.***Total n = 29**

| **Answer option** | **Frequency** | **Percent** |
| --- | --- | --- |
| Once per day | 2 | 6,9 |
| Twice per day | 11 | 37,9 |
| Three times per day | 14 | 48,3 |
| Other (please specify) | 9 | 31,0 |

Specification of other:
6 times per day i small dose

Mixed in the formula or milk given to main meal containing natural protein

not our advise

Four times per day

The geneticist will prescribe this

as per MD

not sure, prescribed by MD

per physician

As per doctor

**Supplemental Table 28 – question 38**

How do you advise I-arginine supplement is given? *Note: multiple answers are possible.*

**Total n = 29**

| **Answer option** | **Frequency** | **Percent** |
| --- | --- | --- |
| With natural protein foods | 7 | 24,1 |
| With low-lysine protein substitute | 5 | 17,2 |
| As a medicine | 20 | 69,0 |
| Other (please specify) | 2 | 6,9 |

Specification of other:

The geneticist will prescribe this
per physician

**Supplemental Table 29 – question 39**Do you advise giving an emergency regimen? *Note: select the answer that applies the most***Total n = 40**

| **Answer option** | **Frequency** | **Percent** |
| --- | --- | --- |
| No | 17 | 42,5 |
| Yes | 12 | 30,0 |
| Some | 8 | 20,0 |
| Not answered | 3 | 7,5 |

**Supplemental Table 30 – question 40.** Why do you advise giving an emergency regimen? *Note: multiple answers are possible.***Total n = 20**

| **Answer option** | **Frequency** | **Percent** |
| --- | --- | --- |
| To prevent catabolism | 16 | 80,0 |
| To prevent breakthrough seizures | 13 | 65,0 |
| Other (please specify) | 3 | 15,0 |

Specification of other:

I am not sure about the neurologists emergency plan
Unsure
We double pyridoxine to prevent breakthrough seizures

**Supplemental Table 31 – question 41**What emergency regimen is prescribed? *Note: multiple answers are possible.***Total n = 20**

| **Answer option** | **Frequency** | **Percent** |
| --- | --- | --- |
| Double dose pyridoxine | 11 | 55,0 |
| Glucose polymer solution | 5 | 25,0 |
| Stop natural protein temporarily | 3 | 15,0 |
| Decrease natural protein temporarily | 6 | 30,0 |
| Aim for usual daily energy intake | 8 | 40,0 |
| Aim for more than usual daily energy intake | 4 | 20,0 |
| Other nutritional requirements (please specify) | 5 | 25,0 |
| Aim for usual intake of lysine-free protein substitute | 9 | 45,0 |

Specification of other:

Aim for regular meals, give KH rich foods and drinks.
we only have an infant so we would say come to ER for assess
Do not know
Aim for more than fluid TFI and DRI protein
CHO containing beverages-gatorade/juice

| **General comments at the end of the questionnaire (question 42)** If results can be shared it would be great Thank you. I look forward to see the result of the questionnaire We haven't experience! This is the first patient with this disease I have also initiated the lysine restricted diet with amino acid supplements in one patients who is not treated in our centre anymore. For this patient (age range 1-12y old) the dietary treatment was stopped by the family because it was too difficult to maintain. This has been completed by the dietitian & the consultant jointly. Thank you for asking me to participate. Catherine It was a bit hard to answer because I have limited experience with just the one patient! |
| --- |
