# Supplementary material for: Dietary management for pyridoxine‐dependent epilepsy due to α‐aminoadipic semialdehyde dehydrogenase deficiency, a follow‐on from the international consortium guidelines
Source: JIMD Rep. 2024 Apr 3;65(3):188–203. doi: 10.1002/jmd2.12418 (PMC11078710; doi:10.1002/jmd2.12418)
Supplement: Supplementary file 2 — Data S2. Examples and calculations. [file JMD2-65-188-s001.docx]

**Supplemental File 2 – Examples and calculations**

**Table:**

Guideline for calculating daily lysine requirements using a combination of 70% low biological value and 30% high biological value protein foods in comparison with FAO/WHO/UNU 2007, Safe level of protein intake.

| **Age**  **Years** | **Body weight**  **kg *** | **Daily lysine requirement mg/kg/day for age (van Karnebeek 2014) ****  **average value** | **Total daily lysine requirement (mg)**  **mg/kg/day x body weight** | **Daily lysine requirement (mg) as 70% from LBV and 30% from HBV foods = natural protein (g) ***** | **Total natural protein g/kg/day** | **FAO/WHO/UNU**  **2007, Safe level of protein intake**  **g/kg/day** |
| --- | --- | --- | --- | --- | --- | --- |
| 1 | 9 | 65 | 585 | 410 = 10g LBV  176 = 3 HBV | 1.4 | 1.14 |
| 2 | 11.5 | 65 | 748 | 524 = 13 LBV  224 = 3 HBV | 1.4 | 0.97 |
| 3 | 13.9 | 65 | 904 | 633 = 16g LBV  271 = 4g HBV | 1.4 | 0.9 |
| 4 | 16 | 65 | 1040 | 728 = 18g LBV  312 = 5g HBV | 1.3 | 0.86 |
| 5 | 18 | 55 | 990 | 693 = 17g LBV  297 = 4g HBV | 1.2 | 0.85 |
| 6 | 21 | 55 | 1155 | 809 = 20g LBV  346 = 5g HBV | 1.2 | 0.89 |
| 7 | 23 | 50 | 1150 | 805 = 20g LBV  345 = 5g HBV | 1.1 | 0.91 |
| 8 | 26 | 50 | 1300 | 910 = 23g LBV  390 = 6g HBV | 1.1 | 0.92 |
| 9 | 29 | 50 | 1450 | 1015 = 25g LBV  435 = 6g HBV | 1.1 | 0.92 |
| 10 | 32 | 50 | 1600 | 1120 = 28g LBV  480 = 7g HBV | 1.1 | 0.91 |
|  |  |  |  |  |  |  |
| **Female** | | | | | | |
| 11 | 36 | 38 | 1368 | 958 = 24g LBV  410 = 6g HBV | 0.83 | 0.9 |
| 12 | 40 | 38 | 1520 | 1064 = 27g LBV  456 = 7g HBV | 0.8 | 0.89 |
| 13 | 45 | 38 | 1710 | 1197 = 30g LBV  513 = 7g HBV | 0.82 | 0.89 |
| 14 | 50 | 38 | 1900 | 1330 = 33g LBV  570 = 8g HBV | 0.82 | 0.87 |
| 15 | 53 | 37 | 1961 | 1373 = 34g LBV  588 = 8g HBV | 0.79 | 0.85 |
| 16 | 55 | 37 | 2035 | 1425 = 36g LBV  611 = 9g HBV | 0.82 | 0.84 |
| 17 | 57 | 37 | 2109 | 1476 = 37g LBV  633 = 9g HBV | 0.81 | 0.83 |
| 18 | 57 | 37 | 2109 | 1476 = 37g LBV  633 = 9g HBV | 0.81 | 0.82 |
|  |  |  |  |  |  |  |
| **Male** | | | | | | |
| 11 | 35 | 38 | 1330 | 931 = 23g LBV  399 = 6g HBV | 0.83 | 0.91 |
| 12 | 38 | 38 | 1444 | 1010 = 25g LBV  433 = 6g HBV | 0.82 | 0.90 |
| 13 | 43 | 38 | 1634 | 1443 = 29g LBV  490 = 7g HBV | 0.84 | 0.90 |
| 14 | 49 | 38 | 1862 | 1303 = 33g LBV  556 = 8g HBV | 0.84 | 0.89 |
| 15 | 56 | 39 | 2184 | 1529 = 38g LBV  655 = 9g HBV | 0.84 | 0.88 |
| 16 | 60 | 39 | 2340 | 1638 = 41g LBV  702 = 10g HBV | 0.85 | 0.87 |
| 17 | 64 | 39 | 2496 | 1747 = 44g LBV  749 = 12g HBV | 0.89 | 0.86 |
| 18 | 66 | 39 | 2574 | 1801 = 45g LBV  772 = 11g HBV | 0.85 | 0.85 |
| *Median weight from the UK-WHO growth charts girls ages 0-4y, 2-18y and the UK 1990 reference for children aged >1years  ******Daily lysine restriction (mg/kg/d) taken as mid-point of the range for age  *** Low biological protein (LBV) - average estimate of 40mg lysine/g of protein and high biological value protein foods - average estimate of 70mg lysine/g of protein | | | | | | |

**Example of a daily menu plan for an 8-year-old boy, weight 26kg (50^th^ percentile), no protein substitute.**

**Daily lysine requirement** for 7<11y (Table 1) = 35-65mg/kg/day. Use upper range of 65mg/kg/d = 1690mg/day

- If no data tables of lysine content of foods, estimate:
- LBV protein foods to provide average of 40mg lysine per g of protein (Table 2)
- HBV protein as dairy foods to provide average of 70mg lysine per g of protein (Table 2)
- Divide daily natural protein as LBV protein foods (70%) and HBV protein foods (30%)

**Normal energy requirement for age** ~ 67kcal/kg = 1720kcal/day

**Micronutrients** – normal for age, add vitamin and mineral supplement.

**Example Diet and Menu Plan: ratio of LBV to HBV protein foods of 70:30**

- LBV protein foods to provide 70% of 1690mg lysine = 1183mg
- Divide LBV lysine allowance by 40mg = 30g protein
- HBV protein foods to provide 30% of 1690mg lysine = 507mg/d
- Divide HBV lysine allowance by 70mg = 7g protein

| **Meal**  **Time** | **Food/Protein substitute** | **LBV protein (g)*** | **HBV protein (g)*** | **Lysine (mg)** |
| --- | --- | --- | --- | --- |
| **Breakfast** | 42g cereal  40g yogurt  ½ banana | 3 | 2 | 120  140 |
| **Snack** | cereal bar  apple | 2 |  | 80 |
| **Lunch** | 150g cooked pasta with tomato sauce mushrooms, onions, and basil,  60g broccoli  45g peas  50g fromage frais + raspberries | 5  2  3 | 2 | 200  80  120  140 |
| **Snack** | crumpet  butter + jam | 3 |  | 120 |
| **Evening meal** | 165g mashed potato (made into potato cake)  60g cauliflower  70g sweetcorn  tinned peaches  60g yogurt | 3    2  2 | 3 | 120  80  80  210 |
| **Bedtime snack** | warmed pitta bread + olives | 5 |  | 200 |
| **Totals** |  | 30g | 7g | 1690mg |
| **Per kg** |  | 1.4g/kg/d | | 65mg |
| **Aim** |  | 30-32g/day | 6-7g/day | 1690mg/day |
| **Safe level of protein intake (42)** |  | 0.92g/kg/d | |  |

***** A different ratio of HBV to LBV protein foods is possible and offers different food choices. A ratio of 50:50 provides a total protein intake above the recommended safe level of protein intake for age.

**Example of a breastfeeding regimen for a 3-month-old female infant, weight 5.6kg.**

**Dietary aims:**

- **Daily lysine requirement**: 100mg/kg/day = 560mg/day
- **Normal energy requirement for age**: ~ 96kcal/kg
- **Protein equivalent (g/kg/day) depends on volume of lysine-free infant formula**
- **Fluid Requirements:** 160ml/kg/d x 5.6kg = 900ml/day

**Daily plan:**

**Breastmilk (estimated 90mg lysine per 100ml breastmilk)** = 620ml = 100mg lysine/kg, 8g protein, 1.4g/kg/day, Energy = 422kcals, Fluids = 111ml/kg

**Lysine free infant formula (2g protein equivalent per 100ml)** = 900ml – 620ml = 280ml = 5.6g PE = 1g kg/day, Energy = 196kcals, Fluids = 50ml/kg

**Total protein** = 8g natural and 5.6g protein equivalent = 13.6g = 2.4g/kg/day

**Total energy** = 618kcals = 110kcals/kg

**Total fluids** = 161ml/kg

**Administration:** 35ml lysine free infant formula followed by a breastfeed to appetite x 3 hourly x 8 feeds per day.

Additional lysine-free infant formula can be given between feeds if the infant is hungry.

**Example of a bottle-feeding regimen for a 3-month-old female infant, weight 5.6kg.**

**Dietary aims:**

- **Daily lysine requirement**: 100mg/kg/day = 560mg/day
- **Normal energy requirement for age**: ~ 96kcal/kg
- **Protein equivalent will vary depending on volume of lysine free infant formula**
- **Fluid Requirements:** 160ml/kg/d x 5.6kg = 900ml/day

**Daily plan:**

**Standard infant formula (114mg lysine per 100ml)** = 490ml/d = 560mg lysine = 100mg lysine/kg, 6.1g protein/day, 1.1g/kg/day, Energy = 328kcals, Fluids = 88ml/kg

**Lysine-free infant formula** **(2g protein equivalent per 100ml)** = 900 – 490ml = 410ml = 8.2gPE = 1.5g/kg/day, Energy = 287kcals, Fluids = 73ml/kg

**Total protein** = 6.1g natural and 8.2g protein equivalent = 14.3g = 2.6g/kg/day

**Total energy** = 615kcals = 110kcals/kg

**Total fluids** = 161ml/kg/day

**Administration:**

80ml standard infant formula followed by 70ml lysine free infant formula (or more to appetite) x 4 hourly x 6 feeds per day.

Additional lysine-free infant formula can be given between feeds if the infant is hungry.
